# Value of adding the renal pathological score to the kidney failure risk equation in advanced diabetic nephropathy

**DOI:** 10.1371/journal.pone.0190930

**Published:** 2018-01-16

**Authors:** Masayuki Yamanouchi, Junichi Hoshino, Yoshifumi Ubara, Kenmei Takaichi, Keiichi Kinowaki, Takeshi Fujii, Kenichi Ohashi, Koki Mise, Tadashi Toyama, Akinori Hara, Kiyoki Kitagawa, Miho Shimizu, Kengo Furuichi, Takashi Wada

**Affiliations:** 1 Department of Nephrology and Laboratory Medicine, Faculty of Medicine, Institute of Medical, Pharmaceutical and Health Sciences, Graduate School of Medical Sciences, Kanazawa University, Kanazawa, Japan; 2 Nephrology Center, Toranomon Hospital, Tokyo, Japan; 3 Nephrology Center, Toranomon Hospital Kajigaya, Kanagawa, Japan; 4 Okinaka Memorial Institute for Medical Research, Tokyo, Japan; 5 Department of Pathology, Toranomon Hospital, Tokyo, Japan; 6 Department of Pathology, Yokohama City University Graduate School of Medicine, Kanagawa, Japan; 7 Department of Nephrology, Rheumatology, Endocrinology and Metabolism, Okayama University Graduate School of Medicine, Dentistry and Pharmaceutical Sciences, Okayama, Japan; 8 Division of Nephrology, Kanazawa University Hospital, Kanazawa, Japan; 9 Division of Internal Medicine, National Hospital Organization Kanazawa Medical Center, Kanazawa, Japan; Tokushima University Graduate School, JAPAN

## Abstract

**Background:**

There have been a limited number of biopsy-based studies on diabetic nephropathy, and therefore the clinical importance of renal biopsy in patients with diabetes in late-stage chronic kidney disease (CKD) is still debated. We aimed to clarify the renal prognostic value of pathological information to clinical information in patients with diabetes and advanced CKD.

**Methods:**

We retrospectively assessed 493 type 2 diabetics with biopsy-proven diabetic nephropathy in four centers in Japan. 296 patients with stage 3–5 CKD at the time of biopsy were identified and assigned two risk prediction scores for end-stage renal disease (ESRD): the Kidney Failure Risk Equation (KFRE, a score composed of clinical parameters) and the Diabetic Nephropathy Score (D-score, a score integrated pathological parameters of the Diabetic Nephropathy Classification by the Renal Pathology Society (RPS DN Classification)). They were randomized 2:1 to development and validation cohort. Hazard Ratios (HR) of incident ESRD were reported with 95% confidence interval (CI) of the KFRE, D-score and KFRE+D-score in Cox regression model. Improvement of risk prediction with the addition of D-score to the KFRE was assessed using c-statistics, continuous net reclassification improvement (NRI), and integrated discrimination improvement (IDI).

**Results:**

During median follow-up of 1.9 years, 194 patients developed ESRD. The cox regression analysis showed that the KFRE,D-score and KFRE+D-score were significant predictors of ESRD both in the development cohort and in the validation cohort. The c-statistics of the D-score was 0.67. The c-statistics of the KFRE was good, but its predictive value was weaker than that in the miscellaneous CKD cohort originally reported (c-statistics, 0.78 vs. 0.90) and was not significantly improved by adding the D-score (0.78 vs. 0.79, p = 0.83). Only continuous NRI was positive after adding the D-score to the KFRE (0.4%; CI: 0.0–0.8%).

**Conclusions:**

We found that the predict values of the KFRE and the D-score were not as good as reported, and combining the D-score with the KFRE did not significantly improve prediction of the risk of ESRD in advanced diabetic nephropathy. To improve prediction of renal prognosis for advanced diabetic nephropathy may require different approaches with combining clinical and pathological parameters that were not measured in the KFRE and the RPS DN Classification.

## Introduction

Despite advances over the past 20 years in delaying the progression of diabetic nephropathy, it is still a leading cause of end-stage renal disease (ESRD) worldwide and imposes a heavy burden not only on individual patients but also on society [1]. Therefore, it is important to be able to predict the risk of developing ESRD in patients with diabetes and chronic kidney disease (CKD) since it could facilitate earlier intervention and better allocation of medical resources to high-risk patients.

Tangri *et al*. developed the kidney failure risk equation (KFRE) for patients with stage 3 to 5 CKD to identify those at high risk of developing ESRD based on demographic, clinical, and laboratory variables [2]. Without considering the etiology of CKD, the simple version of KFRE uses only four clinical variables (age, gender, estimated glomerular filtration rate (eGFR), and urine albumin/creatinine ratio [ACR]) to identify patients at high risk of developing ESRD with a c-statistic > 0.90 (95% CI, 0.894–0.926; *P*<0.001) and has been validated in more than 30 countries with very similar c-statistics [3]. Use of the KFRE in clinical practice could facilitate decision-making for patients with late-stage CKD.

Hoshino *et al*., on the other hand, developed a pathological score for diabetic nephropathy (D-score), based on a pathological classification of diabetic nephropathy by the Renal Pathology Society (RPS DN Classification) [4], to predict ESRD in patients with diabetic nephropathy [5]. Its predictive value of the 10-year risk of ESRD was good with a c-statistics of 0.931 (95% CI: 0.898–0.965).

It has been unclear whether the KFRE can identify ESRD in patients with diabetic nephropathy who have a much higher risk of end-stage disease than other CKD patients, and whether there is any value in combining pathological information on diabetic nephropathy with the KFRE. Therefore, we performed the present study to assess the performance of the KFRE for predicting ESRD in patients with biopsy proven diabetic nephropathy, to investigate the incremental value of combining the D-score with the KFRE, and to validate a new combined model (KFRE + D-score) for predicting ESRD in these patients.

## Materials and methods

### Study sample

To identify patients with type 2 diabetes, we retrospectively reviewed all renal biopsies performed from 1985 to 2013 at the following four nephrology centers: Toranomon Hospital (Tokyo, Japan), Toranomon Hospital Kajigaya (Kanagawa, Japan), Kanazawa University Hospital (Kanazawa, Japan), and Kanazawa Medical Center (Kanazawa, Japan). The indications for biopsy were (i) renal impairment, (ii) urinary abnormalities such as albuminuria, proteinuria, hematuria, or casts, and (iii) suspected concomitant renal disease with diabetic nephropathy. Patients were excluded if they had protocol renal transplant biopsy, confirmed concomitant renal disease (except for nephrosclerosis), or inadequate tissue for diagnosis. Among 521 biopsies, 493 were performed in patients with diabetic nephropathy. Among them, 296 patients with stage 3 to 5 CKD at the time of biopsy were followed up for at least three months, and these patients were enrolled.

This study was approved by the institutional review boards of Toranomon Hospital, Toranomon Hospital Kajigaya, Kanazawa University Hospital, and Kanazawa Medical Center. The study design, clinical setting, eligibility criteria, variables investigated, and statistical analysis were in conformity with the STROBE Statement [6]. This work was partly supported by a Grant-in-Aid for Practical Research Projects for Renal Diseases from the Japan Agency for Medical Research and Development (grant no: 15ek0310003h0001). The funding source had no role in study design or execution, data analysis, manuscript writing, or manuscript submission. The authors have no conflicts of interest to disclose.

### Clinical characteristics, laboratory data, and pathological classification

Clinical characteristics at the time of biopsy, such as the age, gender, duration of diabetes, BMI, hypertension, and dyslipidemia, were ascertained from the medical records.

Laboratory data at the time of biopsy were also obtained from the medical records, including hemoglobin A1c, serum creatinine, eGFR (calculated by the Modified Diet in Renal Disease study equation for Japanese [7]), and ACR or urine protein/creatinine ratio (PCR).

Renal biopsy specimens were processed for light microscopy, immunofluorescence, and electron microscopy. All biopsies were evaluated with three pathologists according to the RPS DN Classification [4]. If all three pathologists did not make the same evaluation, discussion was held until consensus was reached. Diabetic nephropathy was classified as follows. Class I was glomerular basement thickening and only mild, nonspecific changes on light microscopy. Class II was mild (IIa) or severe (IIb) mesangial expansion without either nodular lesions or global sclerosis in >50% of the glomeruli. Class III was nodular lesions without global sclerosis in >50% of the glomeruli. Class IV was global sclerosis in >50% of the glomeruli. Other pathological findings evaluated were interstitial lesions (interstitial fibrosis & tubular atrophy [grades 0–3] and interstitial inflammation [grades 0–2]) and vascular lesions (arteriolar hyalinosis [grades 0–2] and arteriosclerosis [grades 0–2]).

### Outcome measures

The primary endpoint of this study was ESRD, which was defined as initiation of hemodialysis or peritoneal dialysis, or renal transplantation, or death from uremia. Death unrelated to ESRD was also extracted and loss to follow-up was considered a censoring event.

### Statistical analyses

There were no missing data for age, gender, eGFR, and the D-score. However, 207 of 493 patients had missing ACR data but had PCR data. In these patients, we converted PCR to ACR using a converting formula: ln(*ACR*) = 1.32 × ln(*PCR*) − 2.64 [8]. Differences of clinical, laboratory, and pathological variables between the development and validation cohorts were analyzed by Student’s *t*-test or the Wilcoxon test for continuous variables, while the chi-square test or Fisher’s exact test was used for categorical variables.

The 296 patients were randomized 2:1 to the development cohort of 198 and validation cohort of 98 patients. In the development cohort, the KFRE index, the D-score, and the incremental value of the D-score were assessed with using Cox proportional hazard models to test whether predictors were associated with the primary outcome. Model performance was also assessed with calculating the global chi-square, Akaike information criterion, and Harrell’s c-statistics. Furthermore, the area under the receiver operating characteristic curve, net reclassification improvement, integrated discrimination improvement, integrated sensitivity, and integrated specificity for the 3-year risk of ESRD were investigated in the development cohort. To assess incremental values, we followed the guidelines of Kerr *et al* [9]. Then the performance of the two models (KFRE index, D-score, and KFRE index + D-score) was determined in the validation cohort.

Results are expressed as the mean with standard deviation or the median with interquartile range for continuous data and as percentages for categorical data.

Statistical tests were considered significant at *p*<0.05 (two-sided). All statistical analyses were conducted using Stata version 14.1 (StataCorp LLC, College Station, TX).

## Results

### Clinical profile

The study group comprised four independent cohorts of patients (recruited at Toranomon Hospital, Toranomon Hospital Kajigaya, Kanazawa University Hospital, and Kanazawa Medical Center) with type 2 diabetes and advanced CKD (eGFR <60 mL/min/m^2^) who all had biopsy proven diabetic nephropathy without other renal diseases. A total of 296 patients followed for at least three months were randomized at a 2:1 ratio to two groups, which were a development cohort (n = 198) and a validation cohort (n = 98). The median follow-up period (25^th^-75^th^ percentiles) was similar in both cohorts, being 1.8 (1.0–5.0) years in the development cohort and 2.0 (1.0–3.8) years in the validation cohort (p = 0.89). Table 1 shows the baseline characteristics of the development and validation cohorts at the time of renal biopsy. The two cohorts showed no significant differences of clinical variables such as the age, gender, body mass index (BMI), systolic blood pressure, hemoglobin A1c, total cholesterol, estimated glomerular filtration rate, and ACR.

**Table 1:** Clinical characteristics of the two cohorts.

Characteristic	Development cohort	Validation cohort
	n = 198	n = 98
At renal biopsy:		
…Age (yr)	59 (11)	61 (10)
…Male	72 [143]	69 [68]
…BMI (kg/m2)	23.9 (3.8)	23.4 (3.4)
…sBP (mmHg)	148 (21)	146 (21)
…dBP (mmHg)	80 (13)	79 (13)
…HbA1c (%)	7.3 (1.8)	7.6 (2.1)
…TCho (mmol/L)	5.6 (1.9)	6.0 (2.5)
…eGFR (mL/min/1.73 m2)	35.2 (14.7)	35.9 (15.3)
…CKD stage (%)		
…..Stage 3	64 (127)	59 (58)
…..Stage 4	25 (49)	31 (30)
…..Stage 5	11 (22)	10 (10)
…ACR (mg/g)	490 (185, 1848)	642 (168, 1800)
…Albuminuria category (%)		
…..Normoalbuminuria	7 (14)	6 (6)
…..Microalbuminuria	32 (63)	31 (30)
…..Macroalbuminuria	61 (121)	63 (62)
During the entire follow-up period:		
…ESRD (%)	63 (124)	71 (70)
…Death unrelated to ESRD	4 (8)	2 (2)
During follow-up for 3 years after biopsy:		
…ESRD within 3 years (%)	46 (92)	50 (49)
…Death unrelated to ESRD	0 (0)	0 (0)

BMI, body mass index; sBP, systolic blood pressure; dBP, diastolic blood pressure; HbA1c, hemoglobin A1c; TCho; total cholesterol; eGFR, estimated glomerular filtration rate; ACR, albumin/creatinine ratio.

CKD stages 3, 4, and 5 correspond to eGFR of 30–59, 15–29, and <15 mL/min/1.73 m^2^, respectively.

Normoalbuminuria, microalbuminuria, and macroalbuminuria correspond to an ACR of <30, 30–299, and ≥300 mg/g, respectively.

Data are expressed as the mean (standard deviation), median (25^th^, 75^th^ percentiles), or percentage (number).

### Pathological characteristics

Table 2 shows the pathological characteristics of both cohorts. There were no significant differences between the development and validation cohorts in terms of all pathological variables, including glomerular, interstitial, and vascular lesions.

**Table 2:** Pathological characteristics of the two cohorts.

Characteristic	Development cohort	Validation cohort
	n = 198	n = 98
Glomerular lesions:		
…Class		
…..I	16 (32)	14 (14)
…..IIa	8 (16)	10 (10)
…..IIb	16 (31)	20 (19)
…..III	39 (77)	39 (38)
…..IV	21 (42)	17 (17)
Interstitial lesions:		
…IFTA		
…0	4 (7)	1 (1)
…1	24 (48)	22 (21)
…2	29 (58)	39 (38)
…3	43 (85)	38 (37)
…interstitial inflammation		
…..0	2 (4)	1 (1)
…..1	71 (141)	76 (74)
…..2	27 (53)	23 (23)
Vascular lesions:		
…arteriolar hyalinosis		
…..0	2 (5)	3 (3)
…..1	22 (43)	19 (19)
…..2	76 (150)	78 (76)
…arteriosclerosis		
…..0	8 (16)	9 (9)
…..1	47 (93)	49 (48)
…..2	45 (89)	42 (41)

IFTA, interstitial fibrosis and tubular atrophy. Data are expressed as percentages (numbers).

### Development of equations for the KFRE index and D-score

In the original paper by Tangri *et al*. [2], the KFRE is based on four variables, which are the age (per 10 years), gender, baseline eGFR (per 5 mL/min/1.73 m^2^), and logarithmic urine ACR (mg/g). In this study, we used the KFRE index reported by Lennartz *et al* [10]., with hazard ratios obtained from the development cohort:

KFREindex=(age÷10)×ln(0.85)+(gender)×ln(1.22)+(eGFR÷5)×ln(0.81)+ln(ACR)×ln(1.57)

where gender = 1 for male and 0 for female and ln = natural logarithm.

Based on the Pathological Classification of Diabetic Nephropathy by Tervaert *et al*. [4], a pathological risk score for predicting ESRD (D-score [5]) was calculated for each subject. By summing the products of the beta coefficient and bootstrap-inclusion fraction of clinical parameters and the pathological classification of diabetic nephropathy in Cox proportional hazards analysis, the D-scores for glomerular classes I, IIa, IIb, III, and IV were calculated to be 0, 3, 4, 6, and 6, respectively. In addition, the D-scores for interstitial fibrosis & tubular atrophy of grades 0, 1, 2, and 3 were 0, 7, 9, and 11, respectively, while the scores for interstitial inflammation of grades 0, 1, and 2 were 0, 3, and 4, respectively. Furthermore, the D-scores for arteriolar hyalinosis of grades 0, 1, and 2 were 0, 0, and 3, respectively, while the scores for arteriosclerosis of grades 0, 1, and 2 were 0, 0, and 1, respectively.

### Performance of the KFRE and KFRE+D-score

Table 3 summarizes the performance of the KFRE and the KFRE+D-score. The two models were significant predictors of ESRD in both the development cohort and the validation cohort. The KFRE+D-score showed better performance than the KFRE in terms of the global chi-squared value, Akaike information criterion, and Harrell’s c-statistics, but none of the differences were statistically significant. For example, there were no significant differences in Harrell’s c-statistics for the KFRE and KFRE+D-score models in the development cohort (0.776 vs. 0.790, p = 0.83).

**Table 3:** Performance of the KFRE and KFRE+D-score for predicting the risk of ESRD.

Development Cohort	KFRE	D-score	KFRE + D-score
No. of patients	198	198	198
No. of patients with ESRD	123	123	123
Hazard ratio (95% CI)			
KFRE index	2.69 (2.17–3.34)		2.48 (1.98–3.11)
D-score		1.13 (1.08–1.18)	1.07 (1.02–1.12)
χ2	100.9 (p<0.001)	38.7 (p<0.001)	110.2 (p<0.001)
AIC	1001	1064	994
Harrell’s c-statistics	0.776	0.672	0.79
Validation Cohort	KFRE	D-score	KFRE + D-score
No. of patients	98	98	98
No. of patients with ESRD	70	70	70
Hazard ratio (95% CI)			
KFRE index	2.00 (1.56–2.58)		1.97 (1.50–2.60)
D-score		1.14 (1.06–1.23)	1.10 (1.02–1.17)
χ2	31.1 (p<0.001)	15.2 (p<0.001)	39.1 (p<0.001)
AIC	482	498	476
Harrell’s c-statistics	0.747	0.666	0.753

KFRE, kidney failure risk equation; AIC, Akaike information criterion.

### Predicted risk of ESRD after three years

The risk of ESRD was calculated according to the KFRE as reported in the original article^2^:

$$Predicted\;risk\;of\;ESRD\;=\;1-0.535^{e^{0.989\times (KFRE+2.74)}}$$

where 0.535 is the three-year survival rate of an individual with average covariates in the development cohort, 0.989 is the beta coefficient of the KFRE according to the Cox proportional hazards model, and -2.74 is the average KFRE value in the development cohort.

In addition, the predicted risk of ESRD according to the KFRE+D-score was calculated as follows:

$$Predicted\;risk\;of\;ESRD\;=\;1-0.535^{e^{0.910\times (KFRE+2.74)+0.068\times (D-score-19.5)}}$$

where 0.535 is the three-year survival rate of an individual with the average covariates in the development cohort, 0.910 is the beta coefficient of the KFRE according to the Cox proportional hazards model, 0.068 is the beta coefficient of the D-score according to the Cox proportional hazards model, -2.74 is the average KFRE value in the development cohort, and 19.5 is the average D-score in the development cohort.

### Incremental value of the D-score for predicting ESRD at three years in the development cohort

We assessed the incremental value of the D-score for predicting ESRD at three years in the development cohort from the two-category threshold at a prevalence of 46.5% based on the percentage of patients with events. As shown in Table 4, there was no significant change of net reclassification improvement (NRI) when comparing the KFRE model and the KFRE+D-score model. We also investigated the incremental value of the D-score by using free cut-points. Although NRI_nonevents_ showed no significant change, there was a significant change of NRI_events_ that resulted in a positive overall NRI (0.4%; CI, 0.0 to 0.9). The change of integrated discrimination improvement was similar to that of NRI, but was also not significant (0.02; CI: -0.009 to 0.04). Integrated sensitivity was improved in the KFRE+D-score model, whereas integrated specificity was not improved in the KFRE+D-score model. The actual differences from the KFRE model were small.

**Table 4:** Incremental value of the D-score for predicting ESRD at three years in the development cohort.

Measure	KFRE	KFRE + D-score	Incremental value of the D-score
No. of patients	198	198	
No. of patients with ESRD	92	92	
Two-category NRI with risk threshold of 46.5%			
NRIe			5.4 (0.9 to 10.0)
NRIne			0.9 (-3.5 to 5.4)
NRI			6.4 (-2.7 to 15.4)
Category-free NRI (%)			
NRIe			0.4 (0.2 to 0.6)
NRIne			-0.02 (-0.2 to 0.1)
NRI			0.4 (0.0 to 0.8)
IDI statistics			
IDIe			0.02 (0.00 to 0.03)
IDIne			-0.004 (-0.002 to 0.008)
IDI			0.02 (-0.009 to 0.04)
IS and IP statistics			
IS	0.68 (0.64–0.72)	0.70 (0.66–0.74)	
IP	0.40 (0.35–0.45)	0.39 (0.34–0.44)	
Goodness of fit and AUC statistics			
AUC	0.78 (0.72–0.85)	0.80 (0.74–0.86)	0.019 (p = 0.13)
Goodness of fit	0.49	0.52	

AUC, area under the receiver operating characteristic curve; NRI, net reclassification improvement; NRI_e_, category-free event NRI; NRI_ne_, category-free nonevent NRI; IDI, integrated discrimination improvement; IDI_e_, event IDI; IDI_ne_, nonevent IDI. IS, integrated sensitivity; IP, integrated 1-specificity; All values were corrected by the bootstrap technique with 95% confidence intervals.

**Fig 1:**
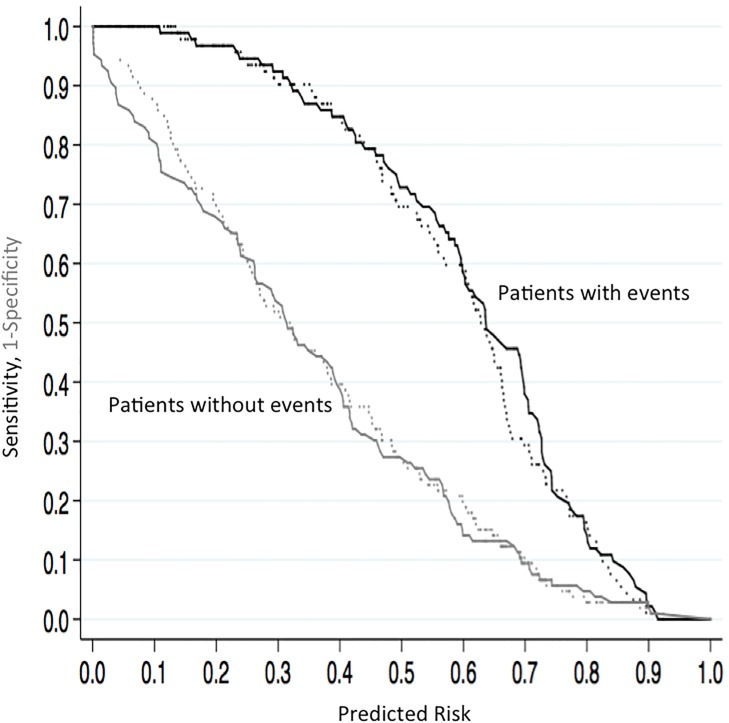
Risk assessment plots with the KFRE model and KFRE+D-score model for predicting ESRD at three years in the development cohort. KFRE model (dashed lines), KFRE+D-score model (solid lines). Black lines indicate sensitivity versus predicted risk. Gray lines represent 1-specificity versus predicted risk.

Fig 1 displays risk assessment plots for the KFRE and the KFRE+D-score. When the incremental value of adding the D-score to the KFRE was assessed, improvement in predicting the risk of ESRD after three years was noted above 0.65 for patients with events and below 0.15 for patients without events.

## Discussion

This study demonstrated that the KFRE and the D-score were independent predictors of ESRD in patients with advanced diabetic nephropathy. However, its predict values were not as good as reported, and combining the KFRE with the D-score did not significantly improve prediction of the risk of ESRD.

A number of authors have proposed prognostic scores for ESRD in patients with advanced CKD, but none of these methods have been widely accepted [11–16]. For more accurate prediction of the risk of ESRD in the clinical setting, Tangri *et al*. developed the KFRE to identify patients with stage 3 to 5 CKD at high risk of progressing to ESRD based on demographic, clinical, and laboratory variables [2]. Without considering the etiology of CKD, the simple version of KFRE employs four clinical variables (age, gender, eGFR, and urine ACR) to identify patients at high risk of ESRD and it has been validated in over 30 countries [3]. However, it has been unclear whether the KFRE performs well in patients who have specific renal diseases associated with a very high risk of ESRD such as diabetic nephropathy. In the present study, we found that the c-statistic of the KFRE was still good, but its predictive value was weaker than that in the miscellaneous CKD cohort originally reported by Tangri *et al*. (c-statistics, 0.78 vs. 0.90). The following points can be given as this reason. First, the KFRE may not be able to capture a phenotype of diabetic nephropathy—nonproteinurics. The simple version of KFRE consists of only four variables—age, sex, estimated GFR, and ACR—and from their hazard ratios we see ACR is the strongest predictor of ESRD among them. However, in our cohort population, we see an increasing frequency of impaired renal function with normoalbuminuria or with microalbuminuria—ACR<300 mg/g (113/296 = 38%)—of our study group. We think this population is a different phenotype of diabetic nephropathy and they are not well captured by KFRE. Second, diabetic nephropathy has a lot of different risk factors of ESRD compared to other CKDs. Other than four variables of KFRE, we have other known risk factors of developing diabetic nephropathy—poor glycemic control, duration of diabetes, hypertension, dislypidemia—and unknown risk factors of developing diabetic nephropathy—race, genetics, and novel biomarkers. Combining these factors may improve prediction of ESRD in patients with diabetic nephropathy. Unfortunately, adding known risk factors did not improve prediction of ESRD with KFRE in our cohort.

For many years, diabetic nephropathy was clinically diagnosed from the presence of macroalbuminuria and renal impairment in patients with diabetes and so there have been a limited number of biopsy-based studies on diabetic nephropathy. Even after Tarvaert *et al*. developed a consensus classification of diabetic nephropathy on behalf of the Renal Pathology Society [4], only a few studies have assessed the prognostic value of this classification [17–19] and none of them investigated the incremental prognostic value of adding renal pathological information to common clinical parameters. The clinical importance of renal biopsy in patients with diabetes, especially in the late-stage CKD, is still debated. In this situation, this study was prepared specifically to clarify the prognostic aspect of renal biopsy in combination with clinical data in advanced diabetic nephropathy. It is important to address this issue because (i) it may change our clinical practice on diabetic nephropathy—we may reduce unnecessary renal biopsy since renal biopsy is an invasive, time-consuming, and costly diagnostic procedure, and (ii) the D-score based on the RPS DN Classification may not include important prognostic pathological parameters and we may be better to seek another unmeasured pathological parameters or clinical parameters that could improve prediction of ESRD in diabetic nephropathy. We found that adding the D-score to the KFRE did not lead to significant improvement in predicting the risk of ESRD. Our findings suggest that we may not perform renal biopsies just for anticipating additional prognostic information from renal pathology based on the RPS DN Classification and that alternatively, to improve prediction of renal prognosis for advanced diabetic nephropathy may require different approaches with combining unmeasured clinical and pathological features.

Despite this result, the main clinical importance of renal biopsy in patients with diabetes is probably related to differentiating diabetic nephropathy from other renal diseases or categorizing concomitant renal pathology. Indeed, several studies have indicated that renal biopsy is useful for differentiating pure diabetic nephropathy from pure non-diabetic nephropathy or combined states since the renal prognosis is different [20–23]. We also think that patients with diabetes and early CKD may gain more benefit from renal biopsy than those with late CKD, since a certain percentage of them develop diabetic nephropathy before renal disease is clinically evident and risk equations like the KFRE based on clinical variables cannot capture this population. This concept was endorsed by Klessens et al. [24] who found biopsy proven diabetic nephropathy at autopsy in 106 out of 168 diabetic patients without a clinical diagnosis of diabetic nephropathy, suggesting that it is considerably underestimated.

Our study had a number of strengths. It was based on a large multicenter cohort of patients with biopsy proven diabetic nephropathy recruited from across Japan. In addition, follow-up was adequate to assess renal outcomes, enabling robust survival analysis of the incremental value combining of renal biopsy data with clinical variables.

However, a number of limitations of this study should be considered. First, there may be a possibility of confounding by indication—the study population was potentially biased by nephrologists who were interested in diabetic nephropathy, since usually it was diagnosed clinically from overt albuminuria and renal impairment in patients with diabetes. Second, the D-score was based on the Pathologic Classification of Diabetic Nephropathy, in which certain pathological changes such as exudative lesions or mesangiolysis, which Furuichi *et al*. [25] reported to be strong predictors of ESRD, were not included. It is possible that adding such pathological information may have improved prediction of ESRD with the KFRE. Third, approximately 40% of data for ACR were not directly measured. They were converted from PCR, using a converting formula for Japanese CKD patients [8]. However, this formula was developed from Japanese CKD cohort and we believe that applying this formula to our Japanese cohort sounds natural. Finally, our study population was limited to Asian patients with type 2 diabetes and advanced CKD, so our findings may not be widely generalizable.

In conclusion, the kidney failure risk equation was a good instrument for identifying a high risk of progression to ESRD among patients with diabetic nephropathy and advanced CKD. However, its predictive value was weaker than in the miscellaneous CKD cohort originally reported by Tangri et al and adding pathological information based on the Diabetic Nephropathy Classification by the Renal Pathology Society to the KFRE did not significantly improve prediction of ESRD. Accordingly, to improve prediction of renal prognosis for advanced diabetic nephropathy may require different approaches with combining unmeasured clinical or pathological features.
